# Pathogenic germline variants are associated with poor survival in stage III/IV melanoma patients

**DOI:** 10.1038/s41598-020-74956-3

**Published:** 2020-10-19

**Authors:** Lauren G. Aoude, Vanessa F. Bonazzi, Sandra Brosda, Kalpana Patel, Lambros T. Koufariotis, Harald Oey, Katia Nones, Scott Wood, John V. Pearson, James M. Lonie, Melissa Arneil, Victoria Atkinson, B. Mark Smithers, Nicola Waddell, Andrew P. Barbour

**Affiliations:** 1 The University of Queensland Diamantina Institute, The University of Queensland, Woolloongabba, QLD 4102 Australia; 2 QIMR Berghofer Medical Research Institute, Herston, QLD 4006 Australia; 3 Division of Cancer Services, Princess Alexandra Hospital, Woolloongabba, QLD 4102 Australia; 4 Queensland Melanoma Project, Princess Alexandra Hospital, Woolloongabba, QLD 4102 Australia; 5 Faculty of Medicine, University of Queensland, St Lucia, QLD 4067 Australia

**Keywords:** Cancer genetics, Cancer genomics, Melanoma, Tumour biomarkers

## Abstract

Patients with late stage resected cutaneous melanoma have poor overall survival (OS) and experience irreversible adverse events from systemic therapy. There is a clinical need to identify biomarkers to predict outcome. Performing germline/tumour whole-exome sequencing of 44 stage III/IV melanoma patients we identified pathogenic germline mutations in *CDKN2A*, *CDK4*, *ATM*, *POLH*, *MRE11A*, *RECQL4* and *XPC*, affecting 7/44 patients. These mutations were associated with poor OS (*p* = 0.0082). We confirmed our findings in The Cancer Genome Atlas (TCGA) human skin cutaneous melanoma cohort where we identified pathogenic variants in 40/455 patients (*p* = 0.0203). Combining these cohorts (n = 499) further strengthened these findings showing germline carriers had worse OS (*p* = 0.0009). Additionally, we determined whether tumour mutation burden (TMB) or BRAF status were prognostic markers of survival. Low TMB rate (< 20 Mut/Mb; *p* = 0.0034) and BRAF *p*.V600 mutation (*p* = 0.0355) were associated with worse progression-free survival. Combining these biomarkers indicated that V600 mutant patients had significantly lower TMB (*p* = 0.0155). This was confirmed in the TCGA (n = 443, *p* = 0.0007). Integrative analysis showed germline mutation status conferred the highest risk (HR 5.2, 95% CI 1.72–15.7). Stage IV (HR 2.5, 0.74–8.6) and low TMB (HR 2.3, 0.57–9.4) were similar, whereas BRAF V600 status was the weakest prognostic biomarker (HR 1.5, 95% CI 0.44–5.2).

## Introduction

The development of cutaneous melanoma is heavily associated with ultraviolet radiation. This environmental influence makes melanoma the most highly mutated cancer type^1^. A subset of patients harbor germline mutations which increases their susceptibility^2^. The predominant high-risk familial melanoma genes are *CDKN2A* and *CDK4*^3–5^. Pan-cancer analysis of 10,389 patients from The Cancer Genome Atlas (TCGA) reported approximately 8% of cases, across 33 cancer types, carried a pathogenic predisposition variant^6^. Importantly, they identified shared variants and genes across several cancer types. Few studies have addressed the clinical impact of pathogenic germline mutations on melanoma patients^7,8^.

The somatic landscape of cutaneous melanoma has been well characterized^9^. BRAF p.V600 is the most commonly mutated coding hotspot present in approximately 40% of patients^9,10^.

Advances in medical research have led to the development of targeted BRAF and MEK inhibitors (BRAFi/MEKi) used as standard treatments for stage IV patients with BRAF p.V600 mutations^11–15^. Recently, adjuvant BRAFi/MEKi have demonstrated a survival benefit for resected stage III patients^16^, however, relapse after complete response to BRAFi/MEKi is common. Another therapeutic option is immunotherapy^17,18^. For BRAF wild-type stage IV patients, immunotherapy is first line treatment^19^ and up to 50% of patients achieve 5-year survival^20^. However, a high proportion of stage III/IV patients have early disease progression and poor survival. Biomarkers are needed to identify patients who benefit from existing therapy and those in need of novel approaches.

Tumour Mutational Burden (TMB) is a somatic biomarker proposed to predict response to immunotherapies in cancer^21,22^. Melanomas can harbor a high TMB^23^ with clinical studies reporting a linear correlation with high TMB and favorable outcomes to PD-1/PD-L1 and CTLA-4 blockade^24,25^. TMB is most predictive in cancers with a high mutation burden and is associated with clinical efficacy of immunotherapies in solid tumour cancers^24,26–30^. To date there is no consensus on the TMB cut-off that best predicts response or prognosis.

In melanoma, Mar et al. described the correlation between the presence of a BRAF/NRAS mutation and a low mutation load^23^. Their study separated BRAF p.V600E and p.V600K mutations. In the clinic, all patients with p.V600 mutations are eligible for targeted therapies regardless of the amino acid change, p.V600E/K/D/R. Collectively, these are class 1 mutations which are the most effective targets of BRAFi/MEKi therapy^14^.

The aim of our study was to determine genomic biomarkers as adverse prognostic factors in 499 melanoma patients. Candidate genomic biomarkers for rapid clinical translation may be germline or somatic^31,32^, therefore we focused on loss-of-function germline mutations in cancer predisposition genes, as well as somatic biomarkers: BRAF mutation status and TMB. We used whole-exome sequencing (WES) data from a Queensland cohort as well as an independent cohort from The Cancer Genome Atlas Program (TCGA) to confirm our findings.

## Results

### Melanoma patient cohort

We conducted a prospective study of late stage cutaneous melanoma patients with completely resected tumours (Table 1). The cohort had a median age of 60 years (range 28–88 years). Thirty-seven patients (stage III, n = 35; stage IV, n = 2) underwent lymph node dissection from which research samples were acquired. For the remaining 7 stage IV patients, tumours were sampled from: omentum, small intestine, gastric, ovary, intransit lesion as well as 2 subcutaneous lesions (Table S1). WES was performed on these tumours as well as the matching blood. The median overall survival (OS) was 27.4 months (range 3.5–50.2 months). The median progression-free survival (PFS) was 9.1 months (range 0.5–47.0 months). The median follow up time for survivors was 32.0 months (range 13.2–50.2 months).

**Table 1:** Clinical characteristics of the study cohort.

Variables	Number of patients (total n = 44)	Percent (%)
**Age at diagnosis (years)**		
< 60	22 (range 28–59)	50.0
≥ 60	22 (range 60–85)	50.0
**Gender**		
Male	30	68.2
Female	14	31.8
**AJCC Stage (8th edition)**		
IIIB	18	40.9
IIIC	16	36.4
IIID	1	2.3
IV	9	20.5
**BRAF p.V600E/K status**		
Mutant	16	36.4
Wild type	25	56.8
Other	3	6.8

### Characterization of germline mutations

To determine whether germline variants were associated with survival, we analyzed 166 cancer predisposing genes (Table S2). In our cohort (n = 44), we identified 11 deleterious germline variants in 10 patients. Six of these were loss-of-function variants that are predicted to truncate the resultant protein.

Two patients had germline mutations in high-risk melanoma genes described as pathogenic in ClinVar and in the literature^2,4^. (Table 2). MelR054 carried a heterozygous missense variant CDK4 p.R24C. MelR191 had a heterozygous CDKN2A c.-34 g > t which leads to aberrant ATG translation at the initiation codon. This results in a truncated protein and decreases translation from the wild-type ATG^33,34^. Four patients had mutations at hotspots in MITF p.E318K (MelR06, MelR191) and TYR p.T373K (MelR014, MelR219) (Table 2). These variants are pathogenic in ClinVar and confer a more modest-risk of developing melanoma^4,35,36^.

**Table 2:** Germline mutations in the Queensland study cohort. Abbreviations: *MAF* minor allele frequency; *AD* autosomal dominant; *AR* autosomal recessive; *BCC* basal cell carcinoma; *LB* likely benign; *LP* likely pathogenic; *NHL* non-Hodgkin’s lymphoma; *P* Pathogenic; *RF* risk factor; *SCC* squamous cell carcinoma.

Sample	Gene	Variant	Consequence	Chr: coordinate	Transcript	rsID	MAF gnomAD	ClinVar Interpretation	ClinVar Calls (no. of submitters)	OMIM	Inheritance
MelR054	CDK4	p.R24C	missense	12:58,145,431	NM_000075.3	rs11547328	0.000004	Pathogenic	P (5), LP (3), RF (1)	Cutaneous malignant melanoma	AD
MelR191	CDKN2A	c.-34 g > t	premature start	9:21,974,860	NM_001363763.2	rs1800586	0.000089	Pathogenic	P (6)	Cutaneous malignant melanoma; Pancreatic Cancer; Neural system Tumour Syndrome	AD
MelR082	ATM	p.H2555_ T2556 delinsQ*	deletion/ insertion	11:108,202,641	NM_001351834.2	rs1555124503	-	Pathogenic	P (2)	Ataxia-telangiectasia (NHL, Leukemia, Hodgkin lymphoma); Breast cancer	AR/AD
MelR162	POLH	p.D74_ L75del	In frame deletion	6:43,550,828	NM_001291969.2	rs1426687865	-	Pathogenic	P (1)	Early onset skin cancer (melanoma, BCC, SCC)	AR
MelR049	MRE11A	p.R364*	stop gained	11:94,200,987	NM_005591.3	rs371077728	0.000067	Pathogenic	P (3)	Ataxia-telangiectasia-like disorder (breast and ovarian cancer)	AR
MelR158	RECQL4	c.2464 -1G > C	splice acceptor	8:145,738,522	NM_004260.3	rs398124117	0.000005	Pathogenic	P (2)	BCC, SCC, Osteogenic sarcoma	AR
MelR041	XPC	p.R579*	stop gained	3:14,199,648	NM_004628.4	rs121965088	0.000012	Pathogenic	P (2)	Early onset skin cancer (melanoma, BCC, SCC)	AR
MelR191	MITF	p.E318K	missense	3:70,014,091	NM_001354604.2	rs149617956	0.001365	Conflicting interpretations of pathogenicity	P (4), LP (2), LB (1)	Susceptibility to cutaneous melanoma	AD
MelR061	MITF	p.E318K	missense	3:70,014,091	NM_001354604.2	rs149617956	0.001365	Conflicting interpretations of pathogenicity	P (4), LP (2), LB (1)	Susceptibility to cutaneous melanoma	AD
MelR014	TYR	p.T373K	missense	11:88,961,072	NM_000372.5	rs61754388	0.000354	Pathogenic	P (7)	Susceptibility to cutaneous melanoma	AD
MelR219	TYR	p.T373K	missense	11:88,961,072	NM_000372.5	rs149617956	0.000354	Pathogenic	P (7)	Susceptibility to cutaneous melanoma	AD

Five patients had pathogenic heterozygous germline mutations in genes predisposing to other cancer types (Table 2). XPC p.R579* was identified in MelR041, ATM p.H2555_T2556delinsQ* was found in MelR082, RECQL4 splice acceptor variant (c.2464-1G > C) in MelR158 and POLH in-frame deletion (p.Asp74_Leu75del) in MelR162. Additionally, MelR049 carried nonsense mutation MRE11A p.R364*, conferring a risk of breast/ovarian cancer^37–39^. These four genes, POLH, MRE11A, RECQL4 and XPC, are autosomal recessive. They are involved in DNA damage repair pathways and have a very broad impact. We have included these loss-of-function mutations in the analysis as these genes are in pathways that have the potential to undergo a 2^nd^ hit which may contribute to tumor development. Mutations in the DNA damage repair genes increase the risk of subsequent mutations and therefore confer high cancer susceptibility.

### Germline mutations and survival

We performed survival analyses (Mantel-Cox) to determine the prognostic significance of germline mutations (Table 2). We excluded MITF p.E318K and TYR p.T373K from our analysis as they are associated with moderate-risk melanoma susceptibility. They are involved in the differentiation of melanocytes/melanoma cells.

**Figure 1:**
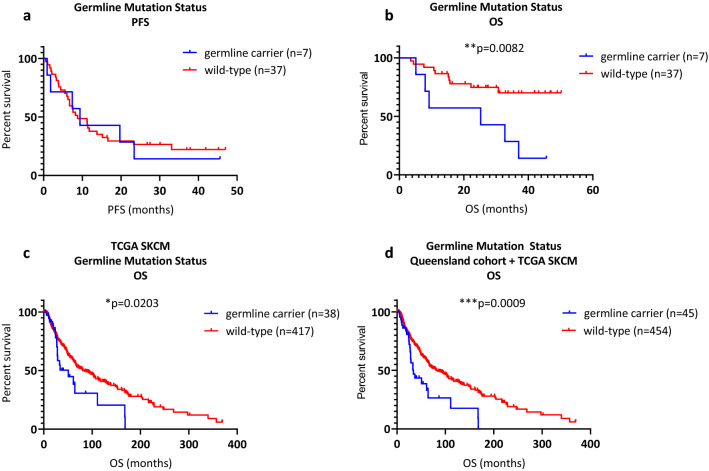
Protein-truncating germline mutations in cancer genes. (**a**) Germline mutation status and PFS, not significant. (**b**) Germline mutation status and OS, **p = 0.0082. (**c**) Germline mutation status and OS in the TCGA SKCM cohort, *p = 0.0203. (**d**) Germline mutation status and OS in the combined Queensland and TCGA cohort, ***p = 0.0009.

We first compared the PFS of variant carriers (median PFS 9.4 months) to wild-type cases (median PFS 8.8 months). We found no significant difference between the two groups (*p* = 0.8171, log-rank, Fig. 1a), indicating that disease progressed at the same rate. However, germline carriers had significantly worse OS (median 25.2 months, *p* = 0.0082, log-rank) than wild-type patients (median survival not reached, Fig. 1b). Three-year OS was 28.6% for carriers compared with 73.0% for wild-type patients.

### Germline mutations in an independent TCGA cohort

The association between high-risk cancer predisposition genes and poor OS was confirmed in an independent cohort from the TCGA human skin cutaneous melanoma (SKCM, n = 455). The median OS was 36.9 months (range 0.2–369.65 months) with a median follow up for survivors of 38.0 months (range 0.2–369.65 months). The OS is not statistically different to our cohort (*p* = 0.0887, log-rank, data not shown). We identified 46 deleterious mutations in 45 individuals. One patient had a mutation in high-risk melanoma gene, CDKN2A p.W15*. We also observed 7 missense mutations attributing a moderate-risk of melanoma (MITF p.E318K, n = 6; TYR p.T373K, n = 1).

Thirty-nine deleterious variants (in 38 individuals) were identified in cancer predisposition genes (Table S3). Several genes were mutated in more than one person: *ATM* (n = 2), *BRCA2* (n = 4), *CHEK2* (n = 4), *FANCC* (n = 2), *MUTYH* (n = 2), *NBN* (n = 2), *RECQL4* (n = 2), SBDS (n = 4), *SDHA* (n = 3), *WRN* (n = 3), and *XPA* (n = 2). Truncating mutations were observed in other genes in single instances: *APC*, *BARD1*^40^, *BLM*, *BRIP1*, *ERCC3*, *MTAP*^41^, *NTHL1*^42^ and *RAD51C*.

In line with the Queensland cohort analysis, we excluded moderate-risk mutations (MITF p.E318K, TYR p.T373K*)*. We performed univariable OS analyses (Mantel-Cox) to compare variant carriers to wild-type patients. Germline variants conferred significantly worse OS (median 50.9 months vs 81.1 months, *p* = 0.0203, Fig. 1c).

Survival analysis (Mantel-Cox) combining the TCGA and the Queensland cohorts (n = 499) further strengthened the findings showing carriers had worse OS than the wild-type group (33.9 months vs 91.14 months, *p* = 0.0009, Fig. 1d).

### BRAF and survival

We next determined whether somatic biomarkers had prognostic significance. Aside from the American Joint Committee on Cancer (AJCC) staging system, BRAF status is the only biomarker commonly used to guide clinical treatment. In our cohort, we confirmed BRAF status using WES. In line with pathology reports, 16 patients harbored a p.V600E/K mutation (p.V600E, n = 14; p.V600K, n = 2) and 3 patients had an alternate mutation: p.L601E, p.T599I and p.L584F.

**Figure 2:**
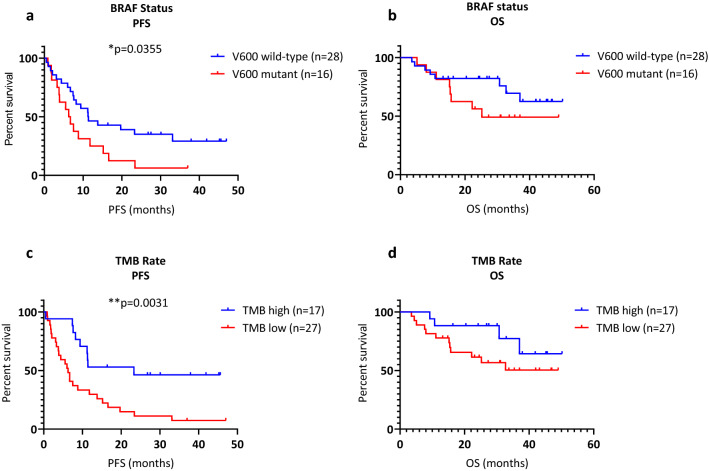
Somatic biomarkers. (**a**) PFS and BRAF status, *p = 0.0317. (**b**) OS and BRAF status, not significant. (**c**) TMB rate and PFS, **p = 0.0034. (**d**) TMB rate and OS, not significant.

Univariable survival analysis showed that V600 wild-type patients had better PFS than V600 mutant patients (median 11.4 months vs 6.5 months, *p* = 0.0355, log-rank, Fig. 2a). However, OS analysis was not statistically significant for BRAF status (median 28.8 months vs 24.1 months, *p* = 0.1756, log-rank, Fig. 2b).

### TMB and survival

Previous studies have shown TMB is a predictive biomarker in a variety of cancers^21,22^. In melanoma, patients have been categorized into high/low TMB using thresholds ranging from 4.81 to 43.2 Mut/Mb^21,43,44^. In our study, the mean TMB was 34.5 Mut/Mb (median 14.5 Mut/Mb). Recursive partitioning methods determined the optimal PFS cut off was 19.75 Mut/Mb, therefore we used 20 Mut/Mb to categorize patients into high/low groups. Univariable survival analysis showed that patients with high TMB had a better PFS than patients with low TMB (median 22.3 months vs 6.3 months, *p* = 0.0031, log-rank Mantel-Cox test, Fig. 2c). OS analysis showed a similar trend, however was not statistically significant (median OS 30.1 months vs 24.5 months, *p* = 0.1374, log-rank Mantel-Cox test, Fig. 2d).

### BRAF wild-type tumours are associated with a higher TMB

**Figure 3:**
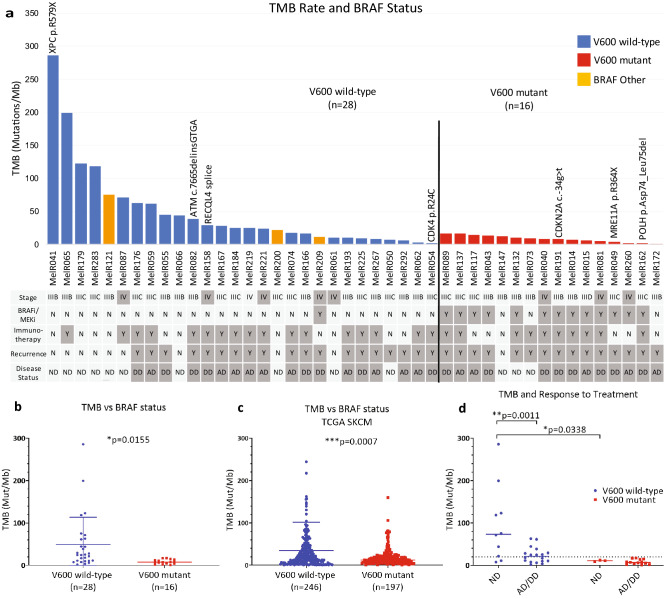
Integrative analysis of germline and somatic biomarkers. (**a**) Histogram detailing the TMB rate in relation to BRAF status. Patients harboring germline mutations are indicated above the bars. Blue bars represent BRAF wild-type. Red bars indicate patients with BRAF p.V600E/K. Orange bars represent patients with other BRAF mutations. Stage, systemic therapy, recurrence and disease status are indicated under the graph. *Y* yes; *N* no; *ND* no evidence of disease; *AD* alive with disease; *DD* dead with disease. Note that MelR050 had surgery upon recurrence of disease. (**b**) BRAF mutation status vs TMB rate in Queensland cohort, *p = 0.0155 and (**c**) TCGA cohort, ***p = 0.0007. (**d**) TMB and disease status. Patients are stratified according to their BRAF status.

We found an association between BRAF status and TMB. V600 wild-type patients (n = 28) had a significantly higher TMB rate (mean 49.3 Mut/Mb) than the V600 mutant group (n = 16, mean 8.5 Mut/Mb) (*p* = 0.0155, two-tailed unpaired t test, Figs. 3a,b). This was confirmed in the TCGA SKCM, where V600 wild-type patients (n = 246) had a higher mean TMB than the V600 mutant group (n = 197), 34.8 Mut/Mb vs. 17.9 Mut/Mb (*p* = 0.0007, Fig. 3c).

### Genomic biomarkers and risk of recurrence

We next determined whether TMB was related to disease recurrence (Fig. 3a). 7/10 patients (9 stage IIIB/C, 1 stage IV) with highest TMB (> 40 Mut/Mb) were disease free (only 2 of these patients received adjuvant immunotherapy). In contrast, 25/27 (92.6%) patients with lowest TMB (< 20 Mut/Mb) had recurrent disease (Figure S1).

Significant differences in disease status were observed when we compared TMB values between V600 mutant and wild-type groups. Focusing on patients who were disease free, the V600 wild-type group had a significantly higher TMB rate (*p* = 0.0338). In BRAF wild-type patients, low TMB was associated with recurrence (*p* = 0.0011, Fig. 3d). V600 mutation carriers had a low TMB and were more likely to recur. In this group, TMB had no predictive power in regards to disease status. Publicly available TCGA data did not include sufficient information on patient treatment and recurrence to be used as a comparable dataset.

**Figure 4:**
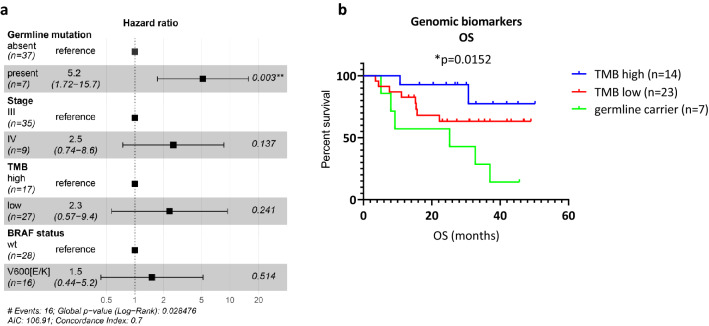
Genomic biomarkers and survival. (**a**) Cox regression analysis of genomic and somatic biomarkers as well as stage. (**b**) OS analysis combining germline and somatic biomarkers, *p = 0.0152. Abbreviation: *wt* wild-type.

We performed a multivariate cox regression analysis integrating stage, germline mutation status, and somatic biomarkers (TMB and BRAF status) with OS. In our cohort, germline mutation status conferred the highest risk (HR 5.2, 95% CI 1.72–15.7; Fig. 4a). AJCC stage IV and low TMB rate had a similar risk (HR 2.5 (95% CI 0.74–8.6) and HR 2.3 (95% CI 0.57–9.4) respectively). BRAF status was the weakest prognostic biomarker (HR 1.5, 95% CI 0.44–5.2) in line with previous observations^10^.

We performed Kaplan Meier OS analysis using the biomarkers with the highest risk: germline mutation status and TMB rate. We first defined germline variant carriers as a distinct sub-group. The remaining patients were classified according to TMB high/low. Analysis showed that the poorest OS was observed in the germline group (*p* = 0.0152, log-rank) while the TMB high patients had the longest survival (Fig. 4b).

## Discussion

We assessed genomic biomarkers as adverse prognostic factors in melanoma in an unselected clinical cohort. In our study, patients have been treated with a variety of therapy combinations. This includes BRAFi/MEKi and/or immunotherapy, which can be given in the adjuvant or palliative setting. Furthermore, a proportion of our patients did not receive systemic therapy and had curative surgery. One of the limitations of this study is that we were not able to assess each therapy individually due to low patient numbers in each group. Our data has shown that the presence of a pathogenic germline variant was the strongest prognostic factor for OS (*p* = 0.0082, n = 44). Germline carriers progressed at the same rate as the non-germline cohort however, once patients relapsed, therapy appeared less effective. This was confirmed in the TCGA SKCM cohort where loss-of-function mutations conferred worse OS (*p* = 0.0203). Notably, TCGA patients were treated as far back as 1977 when most did not benefit from modern systemic therapies. Despite these limitations, the OS was still significantly different.

Germline assessment showed 15.9% (7/44) of the Queensland cohort and 9.9% (45/455) of the TCGA cohort carried a deleterious mutation. The high mutation rate in the Queensland cohort may be attributed to patients having late stage disease which contrasts to the TCGA, comprising all stages. Our observation is in keeping with Mandelker et al. who reported pathogenic variants in 19.7% of patients (205/1040) with advanced cancers (prostate, renal, pancreatic, breast and colon)^45^. Better understanding of the impact of these individual genes on survival would be a very useful clinical tool. For a gene to be included in a genetic test, extensive literature is needed to confirm the consequences of such mutations. Therefore, more studies need to be conducted to validate findings and determine how it can impact clinical practice.

CDKN2A is a well described melanoma predisposition gene. Helgadottir et al. have shown CDKN2A mutation carriers had worse survival than CDKN2A wild-type patients^5^. In a more recent study, Dalmasso et al. reported that CDKN2A mutation carriers had no difference in overall survival outcomes^46^. Although this appears contradictory, this study tested for CDKN2A mutational status at recruitment then patients were assigned to a follow-up scheme accordingly. Germline patients were followed-up more frequently than the wild-type patients. When assessing overall survival, they found no difference between the two groups. This provides further evidence that altering treatment for the germline carriers may improve their overall survival.

Interestingly, MelR041was the only germline carrier (XPC p.R579*) with long OS. This BRAF wild-type patient has the highest TMB rate in our study (285.9 Mut/Mb). They have had multiple primary melanomas (age of onset 23 years) and approximately 50 non-melanoma skin cancers excised. This reflects the clinical features of xeroderma pigmentosum, a condition causing DNA repair defects resulting in photosensitivity and an increased rate of skin cancer^47^. Despite the truncating mutation, the extremely high TMB rate in this patient may contribute to the favorable survival.

When examining TMB rate, we found no correlation between TMB and germline mutation status (data not shown). Further statistical analysis examining the rate of multiple melanomas found no difference in the rate of multiple melanomas between germline carriers and wild-type patients (data not shown). We also analyzed whether germline carriers had a younger age of melanoma onset and found no statistical difference (data not shown). This may be a limitation of our sample size however this observation has been confirmed in the TCGA SKCM by Qing et al.^48^.

We assessed the prognostic value of alternate genomic markers: BRAF status and TMB. BRAF V600 wild-type patients had significantly longer PFS than the V600 mutant group (*p* = 0.0317). This confirms previous clinical studies associating BRAF status with poor OS in stage III patients treated prior to BRAFi/MEKi availability^10,49^. The routine use of targeted therapy in the advanced setting for BRAF mutant patients may explain the improved OS in our study. For stage III/IV resected patients, TMB was also significantly associated with longer PFS (*p* = 0.0034). These results are consistent with recent pan-cancer analysis associating favorable OS with high TMB^50^. This is also reflected in melanoma focused studies^24,51–53^.

Combining BRAF status and TMB, we showed that V600 wild-type patients had a significantly higher TMB rate (our cohort, *p* = 0.0155; TCGA cohort, *p* = 0.0007). This is in concordance with Mar et al. who reported a statistically different tumour mutation rate between BRAF mutant, NRAS mutant and triple wild-type patients (*p* = 0.0004)^23^. In our cohort, NRAS mutant patients (n = 12) were embedded within the wild-type group, in order to reflect the clinical decision-making process.

Using the somatic biomarkers, we assessed risk of melanoma recurrence. V600 wild-type patients with a high TMB rate had low risk of recurrence (*p* = 0.0011). The results showed that for the V600 mutant group, TMB had no predictive power in regards to risk of recurrence post-surgery.

Multivariate survival analysis incorporating the genomic biomarkers with stage found germline mutation status was the most significant biomarker for OS (HR 5.2). When analyzed in parallel with TMB rate, three sub-groups emerged: germline mutation carriers, TMB high patients and TMB low patients. The germline carriers had the shortest OS (*p* = 0.0152) while the longest survival was observed in the TMB high sub-group. Interestingly, all BRAF V600 mutant patients were TMB low. BRAF status is a prognostic factor in other studies^10^. In our cohort, these patients are all TMB low. This raises the question of whether the low TMB status is driving poor survival in this group rather than BRAF status.

In our study, germline status was the most prognostically significant biomarker for OS. Survival outcomes for germline carriers are poor with the current standards of care. Our observations support routine germline testing where mutation carriers should be considered high-risk and put on more intensive follow up.

## Conclusions

We identified protein-truncating germline mutations in cancer genes occurring in 15.9% of late stage melanoma patients. While the treatment of patients is currently based upon BRAF status and AJCC stage, routine germline testing may be incorporated into future iterations of the staging for melanoma to improve prognosis. Combining germline status with BRAF and TMB rate may offer additional tools to stratify patients in the clinic.

## Materials and methods

### Melanoma patient cohort

The study included 44 patients with completely resected stage III/IV melanoma that underwent standard care at the Princess Alexandra Hospital (PAH) Melanoma Unit, Queensland, Australia. Samples were collected at surgery between July 2014 and April 2018. Patients provided informed written consent. Ethics approval was granted by the Metro South Human Research Ethics Committee overseeing research projects at the PAH (HREC/10/PAH/153, HREC/16/PAH/671). All experiments were performed in accordance with approved protocols and regulations. Routine BRAF testing was performed by hospital pathology services. Table S1 details stage, histology and site of primary.

Following surgery, 30 patients received immunotherapy (Pembrolizumab, Ipilimumab, Nivolumab), of these, 20 were V600 wild-type (Table S1). Fourteen patients received BRAFi/MEKi therapy (Dabrafenib, Trametinib, Vemurafenib, Cobimetinib). Ten of these patients also received immunotherapy following progression. MelR209 (stage IV) had BRAF p.L584F and was treated with BRAFi/MEKi. Patients were followed up every 3 months by history and clinical examination as well as CT scan of the head, chest, abdomen and pelvis or whole body PET/CT scan. No patients were lost to follow up.

Tumour DNA was extracted from fresh-frozen tissue stored in RNAlater. Normal DNA was extracted from buffy coat isolated from blood.

### Whole-exome sequencing and SNP array

WES was performed on two platforms. Thirty-three matched tumour/blood samples were sequenced on an Illumina Hiseq4000 using the Agilent sureselect V5 kit. Their overall tumour content was assessed using qpure^54^ by comparing tumour SNP array data (2.5 M Illumina) with the matching normal blood. Eleven matched tumour/blood samples were sequenced on the Illumina NextSeq500 using the IDT pan-cancer spike in. Their cellularity was determined using the mean allele fraction. All samples contained > 20% tumour content. The mean tumour read depth was 439 × (range 254.78–1053.35) for the tumour samples and 216 × (range 52.98–1173.04) for the normal samples (Table S1).

### The cancer genome Atlas data

The TCGA human skin cutaneous melanoma cohort was downloaded and reanalyzed with approval from the QIMR Berghofer Ethics Committee (HREC/P2905). Acral and mucosal samples were excluded. Where patients had more than one tumour sequenced, data from the metastatic site was used. For the germline analysis, 455 patients had sufficient OS data to be included in our study. For TMB and BRAF analysis, patients (n = 443) were included if they had a TMB > 1.0.

### WES analysis

Sequence data was adapter trimmed using Cutadapt v1.9^55^ and aligned to the GRCh37 using BWA-MEM v0.7.15 and SAMtools v1.3^56,57^. Duplicate reads were marked with Picard v2.18.15 MarkDuplicates (https://broadinstitute.github.io/picard). Sample read groups were merged using qbammerge. qProfiler v1.0 and qCoverage v0.7pre performed quality assessment and coverage estimation (sourceforge.net/projects/adamajava).

GATK v3.8 Haplotype Caller and AdamaJava qSNP v2.1.4 were used as a dual calling strategy to detect mutations^58^. GATK v3.8 determined short insertion/deletions (< 200 base pairs) and q3indel v1.0 (AdamaJava) distinguished between somatic and germline calls. All samples had > 100 somatic mutations.

Variants had a minimum 8 reads in the normal data and 12 in the tumour data. Where the variant was identified on both strands, variants required a minimum 4 reads that were not within the first or last 5 bases. The analysis only included rare or novel germline variants with a minor allele frequency < 0.01 in gnomAD.

AdamaJava qannotate v2.1.2 (SNPeff mode) and Ensembl Variant Effect Predictor v92.3 annotated gene and protein consequences^59^.

TMB, reported as mutations per megabase (Mut/Mb) was calculated as a quantitative measurement of all somatic mutations in the coding regions covered by the capture kit.

### Selection of cancer predisposition genes

The germline mutation analysis comprised 166 genes (Table S2). The list included melanoma predisposition genes, cancer predisposition genes reported by COSMIC or the TCGA pan-cancer analysis^6^, as well as genes established through a medical literature review. Additionally, we included genes recommended by The American College of Medical Genetics and Genomics (ACMG)^60^.

Germline variants in this study are pathogenic or likely pathogenic in ClinVar or have strong evidence in the literature to support their pathogenicity.

### Statistical analysis

For the Queensland cohort, overall stage was defined by the AJCC Cancer Staging Manual, 8^th^ edition^61^. A prospectively maintained database (HREC/18/QMS/48,596) provided BRAF status and clinical data. OS was calculated from date of surgery to date of death from disease. PFS was the time from surgery until disease recurrence confirmed through radiology or tumour biopsy.

The Kaplan Meier method was used to analyze OS and PFS (GraphPad Prism 7). Log-rank Mantel-cox tests determined statistical differences between groups. Recursive partitioning defined the optimal high/low cut off for TMB (R Foundation for Statistical Computing). TMB rate between the V600 mutant and V600 wild-type groups, was assessed using an unpaired t-test (two-tailed). Hazard ratio (HR) was determined using a multivariate Cox proportional hazards regression model including established (e.g. tumour stage and BRAF status) as well as proposed biomarkers (TMB and germline mutation status; R Foundation for Statistical Computing).

## Supplementary information

Supplementary Figure.

Supplementary Tables.

## Data Availability

The TCGA dataset is available in the cBioPortal for Cancer Genomics repository (https://www.cbioportal.org/). The dataset from the Queensland cohort are available in the European Genome-Phenome Archive (EGAD00001006374).
