# Supplementary material for: Pathogenic germline variants are associated with poor survival in stage III/IV melanoma patients
Source: Sci Rep. 2020 Oct 19;10:17687. doi: 10.1038/s41598-020-74956-3 (PMC7572377; doi:10.1038/s41598-020-74956-3)
Supplement: Supplementary file 1 — Supplementary Figure. [file 41598_2020_74956_MOESM1_ESM.pdf]

Pathogenic germline variants are associated with poor survival in stage III/IV melanoma patients

Lauren G. Aoude[1], Vanessa F. Bonazzi[1], Sandra Brosda[1], Kalpana Patel[1], Lambros T. Koufariotis[2], Harald Oey[1], Katia Nones[2], Scott Wood[2], John V. Pearson[2], James M. Lonie[1], Melissa Arneil[3], Victoria Atkinson[4,5], B. Mark Smithers[4,5], Nicola Waddell[2], Andrew P. Barbour[1,4]

1. The University of Queensland Diamantina Institute, The University of Queensland, Woolloongabba, QLD, 4102, Australia
2. QIMR Berghofer Medical Research Institute, Herston, QLD, 4006, Australia
3. Princess Alexandra Hospital, Division of Cancer Services, Woolloongabba, QLD, 4102, Australia
4. Princess Alexandra Hospital, Queensland Melanoma Project, Woolloongabba, QLD, 4102, Australia
5. Faculty of Medicine, University of Queensland, St Lucia, QLD, 4067, Australia

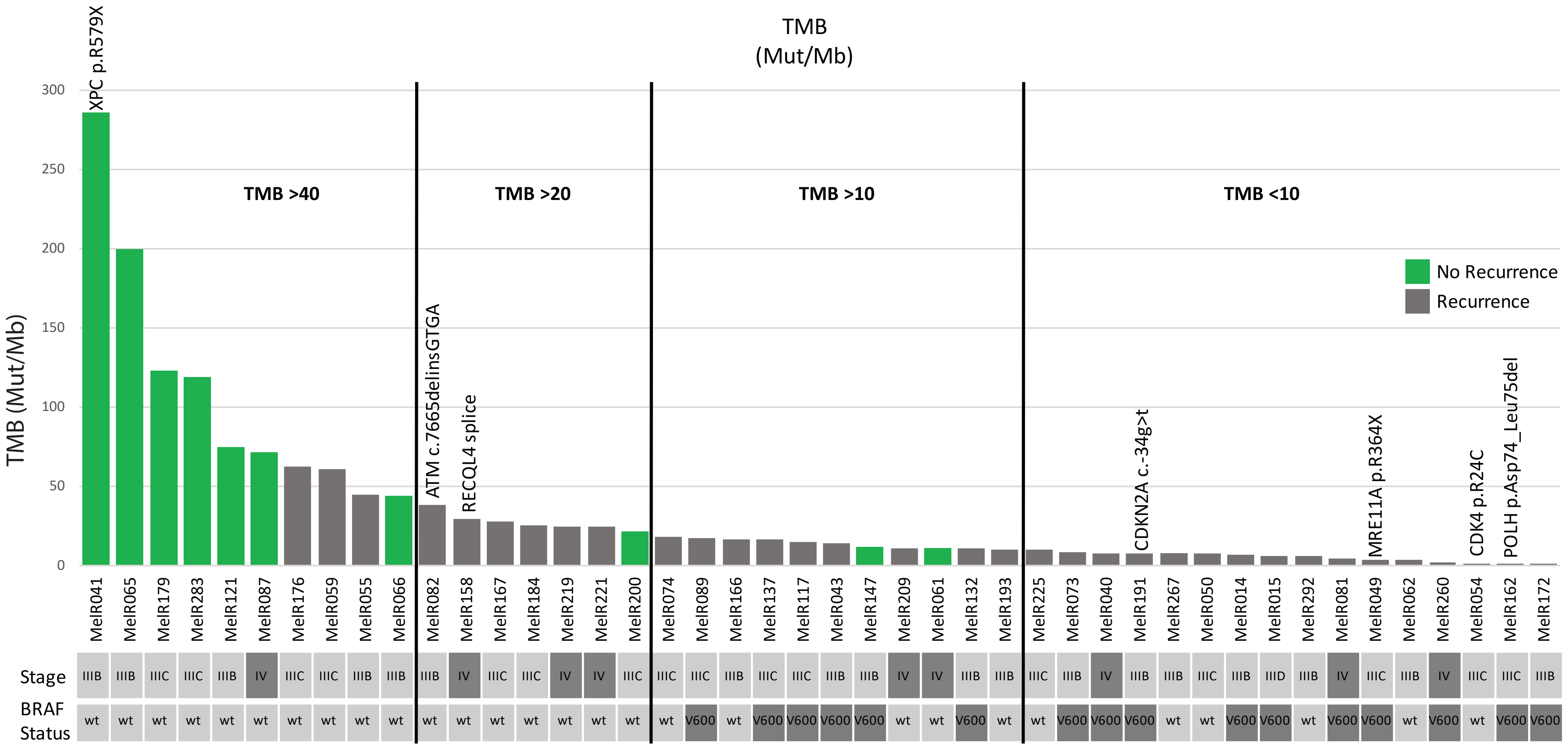

Figure S1. Correlation between TMB and disease recurrence. Patients harbouring germline mutations are indicated above the bars. Green bars represent patients who have not recurred. Grey bars indicate patients who have had melanoma recurrence post-surgery. Stage and BRAF status are indicated below the graph.
